# Estimating a social value set for EQ-5D-5L in Sweden

**DOI:** 10.1186/s12955-022-02083-w

**Published:** 2022-12-23

**Authors:** Sun Sun, Ling-Hsiang Chuang, Klas-Göran Sahlén, Lars Lindholm, Fredrik Norström

**Affiliations:** 1 Department of Epidemiology and Global Health, Umeå University, 90185 Umeå, Sweden; 2 Research Group Health Outcomes and Economic Evaluation, Department of Learning, Informatics, Management and Ethics, Karolinska Institutet, Stockholm, Sweden

**Keywords:** Value set, EQ-5D-5L, Social value

## Abstract

**Background:**

The study aims to elicit a value set based on the EQ-VT for the EQ-5D-5L that can be used to support decision-making in Sweden.

**Methods:**

Participants were recruited from the general population based on age, sex and urban/rural area quota sampling from five regions across Sweden. In total, 785 interviews were conducted from February 2020 to April 2021 using the EQVT 2.1 protocol, and both composite time trade-off (c-TTO) and discrete choice experiments (DCE) were used to elicit health preferences. A variety of models have been tested for the c-TTO data (generalized least square, Tobit, heteroskedastic models) and DCE data (conditional logit model), as well as the combined c-TTO and DCE data (hybrid modelling). Model selection was based on theoretical considerations, logical consistency of the parameter estimates, and significance of the parameters (*p* = 0.05). Model goodness-of-fit was assessed by AIC and BIC, and prediction accuracy was assessed in terms of mean absolute error. The predictions for the EQ-5D-5L health states between models were compared using scatterplots.

**Results:**

The preferred model for generating the value set was the heteroskedastic model based on the c-TTO data, with the health utilities ranging from -0.31 for the worst (55,555) to 1 for the best (11111) EQ-5D-5L states.

**Conclusion:**

This is the first c-TTO-based social value set for the EQ-5D-5L in Sweden. It can be used to support the health utility estimation in economic evaluations for reimbursement decision making in Sweden.

**Supplementary Information:**

The online version contains supplementary material available at 10.1186/s12955-022-02083-w.

## Introduction

Cost-utility analysis has gained popularity in recent years, as it measures both costs and quality-adjusted life years (QALYs) jointly and thus can be applied across different disease areas and intervention programs [1]. In Sweden, QALYs are required for cost-effectiveness analyses by the Health Technology Assessment (HTA) body, in Sweden the Dental and Pharmaceutical Benefits Agency (Tandvårds och läkemedelsförmånsverket in Swedish) in decision making regarding reimbursement of prescription drugs [2, 3].

QALYs are calculated by combining life years and health preferences [4], which reflects an individual’s preference for different health states into a 0–1 scale (where “0” represents “death” and “1” represents “full health”). The commonly applied methods for deriving health preferences are standard gamble [5], time trade-off (TTO) [6], rating scale (RS), ranking exercises and, more recently, discrete-choice experiments (DCE) [7, 8]. However, as measuring health preferences is a rather time consuming and complex task, an alternative approach is to bypass the measurement task by using one of the pre-scored *multi-attribute health status classification systems*, which are also known as the *preference-based measures (PBM)* [7]. PBM consists of a descriptive system and predetermined preference scores known as “value sets” or “tariff”.

The most commonly applied PBMs are the EQ-5D [9], SF-6D [10, 11], and health utilities index (HUI) [12]. The EQ-5D was developed by the EuroQol Group and has been applied widely internationally [13]. The EQ-5D consists of five health dimensions (mobility, self-care, usual activities, pain/discomfort, and anxiety/depression), with each dimension specifying either three (EQ-5D-3L) or five levels (EQ-5D-5L) of severity. The 5L version has been developed more recently to increase the sensitivity and discriminative capacity of the instrument [14, 15].

In Sweden, there has been a tradition of collecting data on Patient-Reported Outcomes (PROs), including the EQ-5D, in many national disease/intervention registries (National Quality Register, NQR) [16]. Since 1998, the Swedish Spine Register and the National Quality Registry for Rehabilitation Medicine were the first NQRs to collect information on the EQ-5D. Since then, the number of registries collecting the EQ-5D has gradually increased over time. Today, 41 out of 105 NQRs are collecting EQ-5D data, 24 on EQ-5D-3L and 17 on EQ-5D-5L. In addition to clinical settings, the EQ-5D instruments have also been applied in population health surveys in several counties [17, 18].

As health systems and how people value health may differ across countries, a country-specific value set is recommended by the EuroQol Group to support decisions regarding resource allocation within the context of a specific country. Country-specific value sets have been established, both for EQ-5D-3L [19] and EQ-5D-5L [20–30]. A variety of survey/interview-based methods can be used to estimate health preferences. Central questions concern whose values to count and which method to use. Two main perspectives can be used, namely, experienced or hypothetical values. The former elicits preferences from individuals who are actually in the health state, and each individual can only value his/her own health state; the latter elicits values from individuals to whom health states are described, and each individual can value a number of health states. Different combinations of health perspectives and methods (SG, TTO, RS, ranking exercises, DCE) may result in different valuation protocols. The protocols developed by the EuroQol Group (http://www.euroqol.org) applied hypothetical values using TTO/VAS (EQ-5D-3L) and *composite TTO* (c-TTO)/DCE (EQ-5D-5L). These protocols are the most widely studied and utilized in eliciting health preferences for the EQ-5D [31]. For the EQ-5D-5L, the *EuroQol Valuation Technology (EQ-VT)* protocol is the standard protocol recommended by the EuroQol Group [32]. The HTA body in Sweden prefers the experience-based value. Thus, the protocol differs from the EuroQol protocols, for both EQ-5D-3L [33] and 5L [34]. To be in line with practice in most other countries, and also to provide opportunities for further investigation regarding whose value to use, it is necessary to establish the hypothetical value for EQ-5D-5L in Sweden as well. The current study will be based on EQ-VT Version 2.1, which is the most recent protocol [35].

## Aim

The aim of this study is to produce a value set based on the EQ-VT for the EQ-5D-5L that can be used to support decision-making in Sweden.

## Method

### The EQ VT valuation protocol version 2.1

The EQ-5D-5L valuation protocol Version 2.1 consists of six sections: 1. Welcome and purpose of the study. 2. Self-reported health using EQ-5D-5L and background questions. 3. c-TTO valuation tasks (including two wheelchair examples to practice the c-TTO technique, three practice states, ten real tasks), debriefing questions, and feedback module where the respondents were asked to review their responses and to flag any health state they felt should be reconsidered. However, those health states could only be flagged but not revalued. 4. DCE valuation tasks (seven tasks, debriefing questions). 5. Comment box; 6. Further background questions. Details of the protocol can be found elsewhere [35].

### The EQ-5D-5L instrument

The official EQ-5D-5L Swedish version provided by the EuroQol Group was applied in the study [36]. The EQ-5D-5L consists of five dimensions: mobility (MO), self-care (SC), usual activities (UA), pain/discomfort (PD), and anxiety/depression (AD). Each dimension has five levels: no problems, slight problems, moderate problems, severe problems, and unable/extreme problems [14]. The combination of different health dimensions and severity levels yields a total of 3125 (${5}^{5}$) unique health states, also known as the ‘health profile’. For example, ‘12,345’ means no problem on MO, slight problems on SC, moderate problem on UA, severe problem on PD and extreme problem on AD. This EQ-5D descriptive system is followed by self-rating of overall health status on a visual analogue scale (EQ VAS) ranging from 0 (‘worst health you can imagine’) to 100 (‘best health you can imagine’).

### Methods for eliciting preferences

There were 86 health states included in the composite time trade-off (c-TTO) task, including the 5 mildest imperfect health states (i.e., 21,111, 12,111, 11,211, 11,121, and 11,112), state 55,555, and 80 other states of varying severity. The 86 health states were grouped into 10 blocks, all of which contained one of the mildest health states, state 55,555, and 8 block-unique health states. The order of health states being shown in each c-TTO block was randomized. Each participant was randomly assigned a health state block for TTO valuation. For DCE valuation, 196 pairs of EQ-5D-5L states were included, following a D-error minimized design[37] and 10 additional pairs comparing between the mildest states [38]. These pairs were divided into 28 blocks of 7 pairs, and each participant evaluated one block. The order of DCE pairs and the left–right position of the health states were randomized.

### Study sample

Data were collected from February 2020 to April 2021 in five regions in Sweden: Västerbotten in the north, Örebro in the middle, Skåne in the south, and Gothenburg and Stockholm representing the most urbanized regions. Members of the Swedish general public aged above 18 were eligible to participate. Quota-based sampling with respect to age, sex and rural/urban area was applied using Swedish population census data [39]. The recruitment strategy engaged leaders in local voluntary organizations, such as pensioner associations, sports clubs and the Red Cross Association, to reach out for participants. Once the potential respondents were targeted, brief information about the study was given by the contact person of each organization. Informed consent was obtained from the respondents before the interview, and detailed information about the study was given in both written and verbal form. For each successful interview, the organization was compensated with 150 SEK (15 Euro).

### Interviewer recruitments and interview process

Initially, 12 interviewers were recruited, all from students at the Karolinska Institute (Stockholm), Umeå University (North), Lund University (South), and Örebro University (Middle). They were all trained at Umeå University in January 2020 following the standard training protocol provided by the EuroQol group. Pilot interviews were performed, and the EQ-VT QC tools were applied to check the quality of the interview. Retraining was conducted when necessary. After evaluating the interview quality, 11 interviewers agreed to conduct field work, which started in February 2020. However, field work was affected by the COVID-19 pandemic and interviews were adapted accordingly through the first months of data collection. To adhere to restrictions on social distance requested by the Swedish government, interviewers were instructed to conduct, face-to-face interviews from mid-June with social distancing (at least two meters distance between the respondent and interviewer given as instruction to interviewers). At the same time, the interviewers were also informed to only conduct already planned interviews and to thereafter take a break due to the pandemic situation. Five interviewers quit the study before the date collection was restarted in September 2020; therefore, four new interviewers were recruited from Gothenburg, trained in August 2020, and two in Umeå one trained in September and one in November. As the pandemic worsened, in late October 2020, it was also approved by the EuroQol Group that video conference could be used instead of face-to-face interviews [40]. We conducted these interviews with via video conference software such as Zoom, requiring the respondent and interviewer to interact through webcam, with the EQ-VT platform shown by shared screen. The study was reviewed and approved by the Swedish Ethical Review Committee (Dnr: 2019–04,950). All data collection was done anonymously, and no other ethical concerns were identified.

### Data quality

Due to the interruption of COVID-19, the turnover of the interviewers was higher than expected. To avoid any potential impact of inexperienced interviewers, it was decided that interviewers with fewer than 10 interviews in the main survey were dropped from the main analysis. Among the rest of the interviewers, the interviewer effect was examined using the *interclass correlation coefficient (ICC)*. The ICC analysis indicated that there was very little interviewer effect, with only 1.3% of variance (95% confidence interval: 0.0036–0.0467) attributed to differences between interviewers.

To understand whether the change in the mode of administration had an impact on the participants’ responses, the data were divided into three categories depending on the date of the survey, cutting off at mid-June and end October 2020, indicating three modes of administration: face-to-face interviews, face-to-face interviews with social distance, and online interviews. A mixed effect model was applied to examine the potential impact, where the mode of administration was used as a fixed effect and respondents nested under interviewers were used as a random intercept. Additional covariates, such as age and gender, were also included (as a fixed effect) in the model to test the impact. The result of the mixed effect model with the mode of administration as a fixed effect and participants nested under interviewers as a random intercept indicated that there was no impact of mode on the c-TTO responses (*p*-value = 0.440). The conclusion remained the same after adding age and sex as fixed effects into the analysis. Thus, all data across the three different modes of administration were pooled together for the primary analysis. We also checked the distributions of c-TTO and DCE scores, time spent on the tasks, which might indicate any potential interviewer effect, and mode of administration due to the COVID-19 pandemic. No statistically significant differences were found.

During the data collection, the quality control (QC) procedure was implemented to evaluate the protocol compliance of the interviewer. The applied QC criteria included completion times, inconsistency size and use of sections of the valuation interview [41]. All included interviewers sufficiently met the criteria for QC, as deemed by the EuroQol group (Additional file 1: Table S1).

### Data analysis and modelling

Descriptive analyses were used to examine the sample characteristics and the responses to the c-TTO and DCE tasks (proportions for discrete variables, mean, and standard deviation for continuous variables). For c-TTO data, those c-TTO health states flagged by the feedback model were also excluded from the modelling analysis. The mean and standard deviation of the c-TTO values were calculated by the level sum score (LSS, the sum of the problem levels in each dimension, ranging from 5 to 25). All DCE data were included in the primary analysis. Statistical modelling was used for the c-TTO (generalized least square (GLS), Tobit, heteroskedastic models) and DCE data (conditional logit model) alone, but also in a hybrid modelling approach combining the c-TTO and DCE data [28]. The choice of modes reflects the characteristics of the data (c-TTO), including the panel structure of the data, a theoretical limit for a worse than dead state at “-1”, and heteroscedasticity of the data. All analyses were conducted in STATA version 14[42].

### Variable and model construction

In the case of c-TTO, the dependent variable was defined as 1 minus the observed c-TTO value for a given health state, indicating the decreased preference score; hence, coefficients expressed health preferences score decrements. For the DCE data, the dependent variable was the binary stated choice (i.e., 0/1 indicated the choice for each health state pair). The dependent variable was explained by 20 dummy variables: four variables for each EQ-5D-5L dimension, each representing level 2 to 5 with level 1 (no problems) as the reference category. No constant term was included in the model. The coefficients presented the decrement from level 1 to the respective level. The regression equation was as follows:

$$\begin{gathered} Y = b1 MO2 + b2 * MO3 + b3 * MO4 + b4 * MO5 + b5 * SC2 \hfill \\ \quad + b6 * SC3 + b7 * SC4 + b8 * SC5 + b9 * UA2 \hfill \\ \quad + b10 * UA3 + b11 * UA4 + b12 * UA5 \hfill \\ \quad +b13 * PD2 + b14 *PD3 + b15 * PD4 + b16 * PD5 \hfill \\ \quad +b17 * AD2 + b18 * AD3 + b19 * AD4 + b20 * AD5 + e \hfill \\ \end{gathered}$$

### Model development and selection criteria

Model selection was based on theoretical considerations, logical consistency of the parameter estimates (i.e., the higher the dimensional level, the higher the utility decrement), and significance of the parameters (*p* < 0.05). Additional information was also provided: model goodness-of-fit was assessed by the Akaike information criterion (AIC) and Bayesian information criterion (BIC); prediction accuracy was assessed for c-TTO models in terms of the mean absolute error (MAE) and root mean squared error (RMSE). The predictions for the EQ-5D-5L health states between models were compared using scatterplots. No comparison was made between models with and without censorship, as they are not comparable.

### Sensitivity analyses

Several sensitivity analyses were conducted. For the c-TTO data, non-traders were excluded from the analysis sample to test their impact. For the DCE data, the potential impact of participants with flat lining behaviour, participants with too short/long responding time, and exclusion 10 pairs of mild-mild health states were examined. Possible combinations of c-TTO and DCE data were also tested in hybrid models.

## Results

### Data

A total of 784 completed interviewers were conducted during the survey, ranging from February 2020 to April 2021. Four interviewers (in a total of nine interview sessions) were excluded since each of the interviewers conducted fewer than 10 interviews. We have included two respondents who valued almost all the health states as 1, as we consider that in a real-life scenario there are people who are known as “non-traders” as they will not trade off life years for better health status [43], and their value should be recognized as well. Thus, 775 interviews were included in the primary analysis. The mean interview time was 49 min, with a standard deviation (SD) of 14 min.

### Characteristics of the respondents

Table 1 presents the characteristics of the analysis sample for both the complete sample (*n* = 784) and the analysis sample (*n* = 775). There were no notable differences that appeared after removal of the nine participants. The proportion of respondents reporting no problem in all five dimensions (health state 11,111) was 30%. The dimension with the most reported problems was pain/discomfort, followed by the anxiety/depression dimension. The mean EQ VAS was 81.0 with a standard deviation of 13.4. Our sample was similar to the Swedish national census data (Additional file 1: Table S2).

**Table 1:** Socio-demographic characteristics and reporting on EQ-5D-5L of the respondents in the Swedish valuation study

Characteristic	Full sample	Analysis sample
	*n*=784	*n*=775
*Socio-demographic characteristics*		
Age, mean (standard deviation)	49.0 (17.6)	49.0 (17.6)
Age group		
18-30	166 (21.2)	166 (21.4)
30-49	228 (29.1)	225 (20.3)
50-65	215 (27.4)	212 (27.4)
>65	175 (22.3)	172 (22.2)
Sex		
Female	470 (60.0)	465 (60.0)
Male	311 (40.0)	307 (39.6)
Other	3 (0.4)	3 (0.4)
*Education *		
Less than 9 years of elementary school	23 (3.0)	23 (3.0)
9 years of elementary school	269 (34.3)	269 (34.7)
High school	8 (1.0)	8 (1.0)
University	484 (61.7)	475 (61.3)
*Occupational status*		
Permanent employed	391 (49.9)	386 (49.8)
Self-employed	51 (6.5)	51 (6.6)
Temporary employed	39 (5.0)	37 (4.8)
Not employed	16 (2.1)	16 (2.1)
Retired	195 (24.9)	193 (24.9)
Student (full-time)	92 (11.7)	92 (11.9)
*EQ-5D-5L descriptive system*		
Mobility		
No problems	658 (83.9)	652 (84.1)
Slight problems	93 (11.9)	90 (11.6)
Moderate problems	27 (3.4)	27 (3.5)
Severe problems	5 (0.6)	5 (0.7)
Unable to walk about	1 (0.1)	1 (0.1)
*Self-care *		
No problems	756 (96.4)	748 (96.5)
Slight problems	25 (3.2)	24 (3.1)
Moderate problems	2 (0.3)	2 (0.3)
Severe problems	1 (0.1)	1 (0.1)
Unable to dress and wash	0 (0)	0 (0)
*Usual activities*		
No problems	641 (81.8)	633 (81.7)
Slight problems	108 (13.8)	107 (13.8)
Moderate problems	29 (3.7)	29 (3.7)
Severe problems	6 (0.8)	6 (0.8)
Unable to do usual activities	0 (0)	0 (0)
*Pain/discomfort*		
No pain or discomfort	333 (42.5)	330 (42.6)
Slight pain or discomfort	342 (43.6)	338 (43.6)
Moderate pain or discomfort	96 (12.2)	94 (12.1)
Severe pain or discomfort	12 (1.5)	12 (1.6)
Extreme pain or discomfort	1 (0.1)	1 (0.1)
*Anxiety/depression*		
No anxiety or depression	481 (61.4)	473 (61.0)
Slight anxiety or depression	253 (32.3)	252 (35.5)
Moderate anxiety or depression	38 (4.9)	38 (4.9)
Severe anxiety or depression	11 (1.4)	11 (1.4)
Extreme anxiety or depression	1 (0.1)	1 (0.1)
VAS, mean (SD)	81.0 (13.3)	81.0 (13.4)
*Self-rated health *		
Very good	313 (40.1)	311 (40.1)
Good	380 (48.5)	375 (48.4)
Fair	82 (10.5)	80 (10.3)
Bad	1 (0.1)	1 (0.1)
Very bad	8 (1.0)	8 (1.0)

### Descriptive analysis of c-TTO and DCE data

**Fig. 1:**
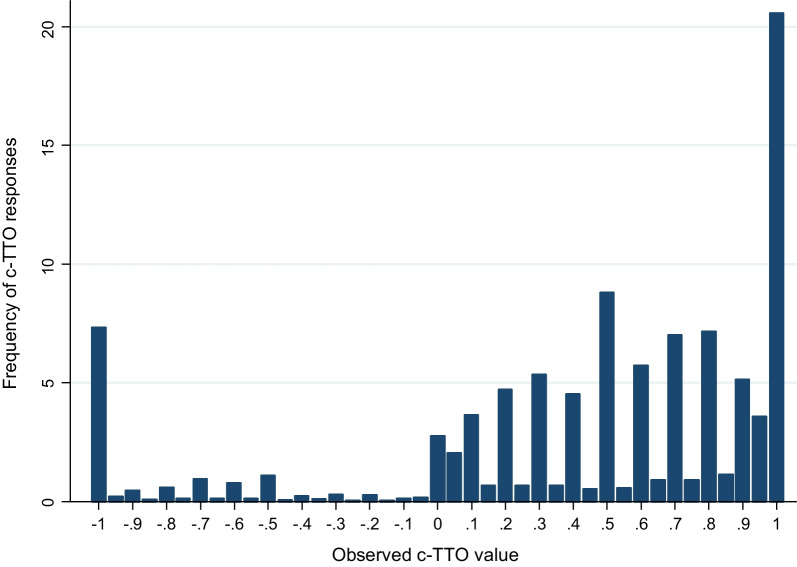
distribution of observed c-TTO value (*n* = 7750)

A total of 7750 c-TTO values, evaluating 86 health states, were collected. The distribution of c-TTO values is presented in Fig. 1. Among all c-TTO responses, 21% of c-TTO health states were valued as 1 and 13% with values less than 0 (worse than dead). Among the respondents, 17% had one logical inconsistency, and 8% had more than 2 logical consistencies. The number of c-TTO values flagged by the feedback module was 876 (11%). After excluding the flagged values, logical inconsistency was reduced to 7% and 2% for one and two logical inconsistencies, respectively. The mean c-TTO values for observed health states ranged from 0.982 for 11,121 to − 0.285 for 55,555. For model construction, all the un-flagged c-TTO responses (*n* = 6874) were used. The mean c-TTO values decreased with increasing LSS, whereas standard deviations increased with LSS (Additional file 1: Figure S1). Of all 6874 responses, 13% had values less than 0 and 22% had a value of ‘1’, and all were used in the primary analysis.

Regarding the DCE task, a total of 5,425 responses collected from 196 DCE pairs were included in the primary analysis. As shown in Additional file 1: Figure S2, respondents were more likely to choose the health state with a lower LSS score when the severity between the two states increased. Responses with flat lining behavior (such as AAAAAAA or ABABABA) were identified. However, they were not excluded from the analysis set (*n* = 22).

### Modelling results and the value set

The modelling results based on c-TTO, DCE and hybrid data are presented in Table 2. All coefficient estimates in c-TTO-based models were logically consistent (coefficient size was larger with a more severe level of problems), while the DCE-based model had two logical inconsistencies in the self-care and usual activities dimensions (both in level 3), and all hybrid-based models had one logical inconsistency in the usual activities dimension (level 3). All c-TTO- and DCE-based models possessed at least one coefficient estimate that was not statistically significant, including mobility, self-care and pain/discomfort dimensions for c-TTO and self-care and usual activities dimensions for DCE, whereas all estimates in hybrid models were statistically significant. Dimension ranking in terms of relative importance (based on the magnitude of the estimate of level 5) differed slightly between models. c-TTO-based models had the most important dimensions of pain/discomfort and anxiety/depression, followed by usual activities, self-care and mobility, while in DCE and hybrid, the order of self-care and mobility was reversed. Among the c-TTO-based models, compared to the GLS and Tobit models, the heteroskedastic model generates smaller estimates for level 2 and 3 (except self-care) in comparison to the other two models. All sensitivity analyses generated the same results (Additional file 1: Table S3).

**Table 2:**
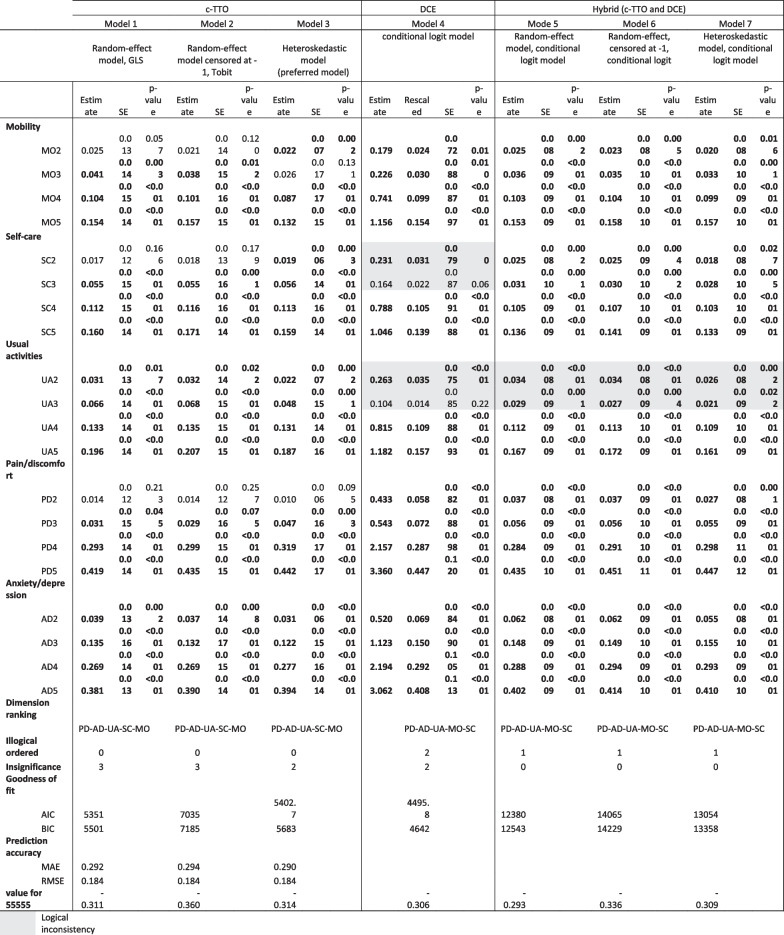
Parameter estimates for main effects models

### The preferred value set

**Fig. 2:**
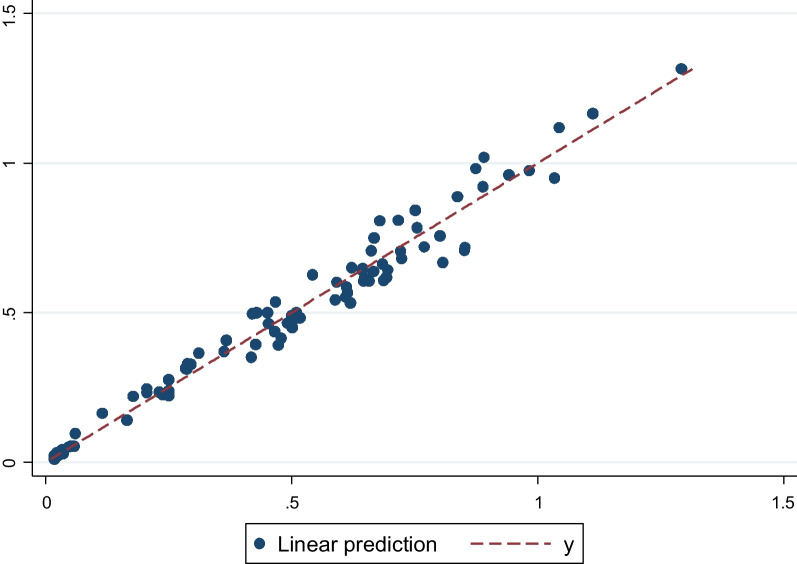
Scatterplots of the predicted values of the heteroskedastic model versus observed values of c-TTO

The heteroskedastic model based on c-TTO data was selected as the preferred model for the value set. Reasons are that DCE and hybrid models suffered more from logical inconsistencies relative to the c-TTO models, plus the DCE models were also associated with rescaling issue as “dead” was not involved in DCE valuation task, for hybrid model it lacks theoretical foundation of combining both DCE and TTO data together. Among the c-TTO models, similar performances were observed in terms of logical consistency and statistical significances. The heteroskedastic model was preferred for its ability to address the variability of the error term and its slightly better significance level than the other two c-TTO models. Furthermore, the scatterplot of predicted values of the heteroskedastic model versus observed values of c-TTO are presented in Fig. 2. It seems that the heteroskedastic model performed slightly better predictions in the higher end. (The figure of the c-TTO GLS model vs observed values of c-TTO is shown in Additional file 1: Figure S3). Its predicted value for state 55,555 is − 0.314.

EQ-5D-5L health state utilities can be estimated by subtracting the relevant decrement for each problem on each dimension from 1. For example, the EQ-5D-5L index health state 12,345 was calculated as following:

$$EQ - 5D_{index } = 1 - 0 \left( {MO1} \right) - 0.019 \left( {SC2} \right) - 0.048 \left( {UA3} \right) - 0.319 \left( {PD4} \right) - 0.394 \left( {AD5} \right) = 0.220$$

## Discussion

This study presents the results of the Swedish EQ-5D-5Lvalue set, based on the EQ-VT 2.1 protocol. The value set can be used to estimate health preferences score based on EQ‐5D‐5L instrument, and can be used to calculate QALYs in the economic evaluation of health care interventions to support resource allocation decisions.

Whose values should be used (hypothetical or experience-based value) is a fundamental question in valuing health states, and it is still an on-going debate [7, 44–46]. Comparative studies have shown that experience-based values tend to be higher than hypothetical values [33, 45, 47–49], and the mental dimensions seem to be more important than physical detentions [34, 46, 48, 50]. The EuroQol Group supports the use of hypothetical values, and their arguments are that health services are funded by taxpayers; therefore, values from a representative group of the general public should be counted. In contrast, the HTA body in Sweden prefer the use of experience-based values; they also stated that “*QALY weightings based on appraisals of persons in the health condition in question are preferred before weightings calculated from an average of a population estimating a condition depicted for it (e.g., the “social tariff” from EQ-5D* [2, 3]. Their arguments are that people in that health condition know about it best, and adaptation shall be reflected in valuation.

Comparing our value set with the Swedish experience-based value set [34], considerable differences were found. For the experience-based value, all health states are considered better than dead, including 55,555 (0.244), and no full health can be reached, as 11,111 is valued as 0.976. For health values based on hypothetical values, 4% (*n* = 138) are associated with negative values and are considered as health states worse than dead, full health can be reached as 11,111 is defined as 1. The mean EQ-5D index based on the experienced value set is 0.605 (SD = 0.14), and that based on the hypothetical value set is 0.471 (SD = 0.26). In general, for mild health states, the experience-based values are lower than the hypothetical values; for more severe health states, the experience-based values are higher. For experience-based values, anxiety/depression usually has the greatest impact on overall health [34, 47, 48]; for hypothetical values, it is pain/discomfort. There might be restricted direct comparability due to the two different type of valuation tasks using completely different methods. However, it is still important to further investigate the implication of whose value to use, i.e., whether they impact the results of cost-effectiveness analyses. If so, will such impact differ across different disease? However, this is not the focus of current study, and should be addressed by future studies.

It is difficult to say which value set to choose, and the choice might depend on the decision-making context. Drummond et al. all recommended that for decisions related to how to allocate public funds to a new intervention, it might be more appropriate to use hypothetical values, whereas decisions regarding existing treatment options that are already funded might be more appropriate to use experience-based values [7]. Therefore, we advocate that for cost-effectiveness studies in Sweden, for the moment both hypothetical and experience value sets shall be applied to adapt to different decision-making contexts. Further research is needed to understand the implication of two different value sets on the estimation of QALY and QALY gains across different patient groups as well as the general population.

We found that the Swedish value set is more similar to those from other western countries/areas, where either anxiety/depression or pain/discomfort are considered as the most important dimensions, while among Asian countries the mobility dimension is considered as the most important dimensions. The scale length (difference between values for 11,111 and 55,555) for the Swedish value set is 1.314, which is similar to other western countries (ranging between 1.096 and 1.757). For the Swedish value set, the midpoint in the value distribution (difference between values for 11,111 and 33,333 divided by scale length) is 22.8%, which is close to the Germany (20.4%), Denmark (24.4%) and Hungary (23.2%) studies [41]. However, such differences may not only be due to differences in health preferences associated with cultural values, wealth and characteristics of health systems, but also be due to differences in measurement method, data quality and modelling strategies [41]. There are significant differences between different versions of EQ-VT protocols (version 1.0, 1.1, 2.0 and 2.1), data included for value set (c-TTO and DCE combined, or c-TTO only) and modelling techniques (the assumption on censoring at -1; how heteroscedasticity is handled; accounting for preferences heterogeneity). Therefore, differences in value set across countries need to be interpreted with caution.

In the current study, the DCE model generated more inconsistency than the c-TTO model. Various sensitivity analyses generated the same results. One might be concerned about the DCE data quality. Unlike the c-TTO data, within the EQ-VT protocol, there are no criteria for evaluating the quality of DCE data [35, 38]. The lack of a DCE procedure or data quality control might be an improvement for future EQ-VT DCE designs. Furthermore, the c-TTO and DCE tasks differ by their nature: in the c-TTO task, health states are valued against time, which is considered a matching task, while in the DCE task, health states that differ in terms of the degree of severity of the dimensions are directly compared with another (choice task)[7, 51]. The usual expectation is that for the respondents, the DCE task may be easier than the c-TTO task. However, in this study, there were more inconsistencies in the DCE model than in the c-TTO model. It may be that the usual perception did not hold in the Swedish context. Further investigations are needed.

In the current study, the use of hybrid models did not remove the problem of inconsistencies as seen in the DCE models, and this is the main reason for not choosing the hybrid model. The lack of a theoretical foundation for combining both TTO and DCE data is another concern. To date, all evidence supporting the use of hybrid models is empirically based, while no theory justifying the mix of two unique data types has been proposed. Specifically, the support for the hybrid approach is based on the apparently high correlation between c-TTO scores and latent utilities derived from DCE responses. Many valuation studies reported correlation coefficients (*r* >  = 0.9) to justify this approach. However, correlation alone is not a sufficient criterion. In addition, a study by Pullenayegum and colleagues shows that, using the multivariate latent class model based on Canadian valuation data, there is poor agreement between TTO and DCE data at the response level [52]. Other studies also show that the linearity of the relationship between TTO and DCE may not hold [53].

Due to the COVID-19 pandemic, throughout the data collection phase, the study had to continuously recruit new interviewers and switch the administration mode from face-to-face interviews to interviews with social distance and finally to online interviews. This also added challenges to reach the goal of including at least 1000 respondents according to the EuroQol’s recommendations [38]. With a larger sample size, we would have greater statistical power, and probably less problems with in-significant coefficients. The small sample size (*n* = 775) must be addressed as a limitation of the current study. However, we expect the size and pattern of the coefficients of the value set to at most differ marginally in relation to current results, considering the similarity of coefficients across different models. Our sample size is similar to the Uruguayan study, which had 794 respondents [54]. Furthermore, the possible effect due to change of interviewers and mode of administration were investigated in the current study, and the results indicated no obvious evidence against pulling all data together. the pandemic itself can have an impact on how people value health, but it is not possible to sort out all these effects. Thus, the impact of the COVID-19 pandemic on data collected for EQ-VT remained a major limitation of the present study.

## Conclusions

This is the first c-TTO-based social value set for the EQ-5D-5L for Sweden. It can be used to support the health utility estimation in economic evaluations for reimbursement decision making in Sweden.

## Supplementary Information

**Additional file 1: Table S1.** Result of QC procedure. **Table S2.** Study sampled compared with the Swedish census data. **Table S3.** Sensitivity analysis results. **Fig. S1**. c-TTO value by level of sum score, after excluded values flagged by feedback model (*n*=6874). **Fig. S2.** Relative preference for health state A versus B by difference in level of sum score for discrete choice experiment tasks. **Fig. S3.** Scatterplots of the predicted values of the GLS model versus observed values of c-TTO.

## Data Availability

Not applicable.
