# Supplementary material for: Estimating a social value set for EQ-5D-5L in Sweden
Source: Health Qual Life Outcomes. 2022 Dec 23;20:167. doi: 10.1186/s12955-022-02083-w (PMC9780618; doi:10.1186/s12955-022-02083-w)
Supplement: Supplementary file 1 — Additional file 1: Table S1. Result of QC procedure. Table S2. Study sampled compared with the Swedish census data. Table S3. Sensitivity analysis results. Fig. S1. c-TTO value by level of sum score, after excluded values flagged by feedback model (n=6874). Fig. S2. Relative preference for health state A versus B by difference in level of sum score for discrete choice experiment tasks. Fig. S3. Scatterplots of the predicted values of the GLS model versus observed values of c-TTO. [file 12955_2022_2083_MOESM1_ESM.docx]

Table S1. Result of QC procedure

| **Interviewer** | **N** | **% flagged** | **% WC LT^1^** | **% Incon size^2^** | **% WC time^3^** | **% TTO time^4^** |
| --- | --- | --- | --- | --- | --- | --- |
| **Interviewer 1** | 117 | 5% | 1% | 0% | 3% | 3% |
| **Interviewer 2** | 211 | 8% | 3% | 2% | 3% | 0% |
| **Interviewer 3** | 16 | 6% | 6% | 0% | 0% | 0% |
| **Interviewer 4** | 51 | 10% | 6% | 4% | 6% | 0% |
| **Interviewer 5** | 56 | 0% | 0% | 0% | 0% | 0% |
| **Interviewer 6** | 12 | 8% | 0% | 0% | 0% | 8% |
| **Interviewer 7** | 52 | 2% | 0% | 0% | 0% | 2% |
| **Interviewer 8** | 22 | 0% | 0% | 0% | 0% | 0% |
| **Interviewer 9** | 83 | 5% | 0% | 1% | 3% | 1% |
| **Interviewer 10** | 111 | 7% | 4% | 3% | 1% | 0% |
| **Interviewer 11** | 44 | 2% | 3% | 0% | 0% | 0% |

1. No explanation of the worse than dead task (lead time) on the wheelchair example. 2. Clear inconsistency in the TTO ratings (33333 / 55555 is not the lowest and at least 0.5 higher than the state with the lowest value). 3. Too short time period spent on the wheelchair example (less than 3 min). 4. Too short time period for the TTO task (total time for the 10 TTO tasks less than 5 min).

Table S2. Study sampled compared with the Swedish census data

|  | Sample used for current study  (Analysis sample) | Swedish population from Census data |
| --- | --- | --- |
| Age group |  |  |
| 18-30 | 21.4% | 16% |
| 30-49 | 20.3% | 34% |
| 50-65 | 27.4% | 24% |
| More than 65 | 22.2% | 26% |
| Men/Female | 39.6%/60%/0.4%(other) | 50%/50% |

Table S3. Sensitivity analysis results

|  | Model 1 | | |  | Model 2 | | |  | Model 3 | | |  | Mode 4 | | |  | Model 5 | | |  | Model 6 | | |
| --- | --- | --- | --- | --- | --- | --- | --- | --- | --- | --- | --- | --- | --- | --- | --- | --- | --- | --- | --- | --- | --- | --- | --- |
|  | DCE: remove flat lining data | | |  | DCE: remove shorter response time (<200 sec.) | | |  | DCE: without 10 mild pairs | | |  | Hybrid: random-effect model, conditional logit model | | |  | Hybrid: random-effect, censored at -1, conditional logit | | |  | Hybrid: heteroskedastic model, conditional logit model | | |
| Dimension/level | Estimate | SE | P value |  | Estimate | SE | P value |  | Estimate | SE | P value |  | Estimate | SE | P value |  | Estimate | SE | P value |  | Estimate | SE | P value |
| **Mobility** |  |  |  |  |  |  |  |  |  |  |  |  |  |  |  |  |  |  |  |  |  |  |  |
| MO2 | 0.182 | 0.070 | 0.01 |  | 0.194 | 0.072 | 0.007 |  | 0.023 | 0.081 | 0.775 |  | 0.016 | 0.009 | 0.078 |  | 0.013 | 0.009 | 0.167 |  | 0.013 | 0.009 | 0.156 |
| MO3 | 0.220 | 0.089 | 0.014 |  | 0.207 | 0.092 | 0.025 |  | 0.158 | 0.092 | 0.085 |  | 0.030 | 0.010 | <0.001 |  | 0.028 | 0.010 | 0.006 |  | 0.027 | 0.010 | 0.007 |
| MO4 | 0.739 | 0.088 | <0.001 |  | 0.724 | 0.090 | <0.001 |  | 0.648 | 0.091 | <0.001 |  | 0.100 | 0.009 | <0.001 |  | 0.100 | 0.010 | <0.001 |  | 0.100 | 0.010 | <0.001 |
| MO5 | 1.164 | 0.101 | <0.001 |  | 1.138 | 0.103 | <0.001 |  | 1.075 | 0.102 | <0.001 |  | 0.146 | 0.009 | <0.001 |  | 0.151 | 0.010 | <0.001 |  | 0.153 | 0.010 | <0.001 |
| **Self-cate** |  |  |  |  |  |  |  |  |  |  |  |  |  |  |  |  |  |  |  |  |  |  |  |
| SC2 | 0.256 | 0.079 | 0.001 |  | 0.195 | 0.080 | 0.015 |  | 0.106 | 0.094 | 0.258 |  | 0.021 | 0.009 | 0.022 |  | 0.020 | 0.010 | 0.038 |  | 0.016 | 0.009 | 0.091 |
| SC3 | 0.164 | 0.088 | 0.062 |  | 0.169 | 0.090 | 0.06 |  | 0.054 | 0.091 | 0.558 |  | 0.027 | 0.010 | 0.006 |  | 0.026 | 0.010 | 0.014 |  | 0.023 | 0.010 | 0.022 |
| SC4 | 0.773 | 0.091 | <0.001 |  | 0.758 | 0.094 | <0.001 |  | 0.694 | 0.094 | <0.001 |  | 0.102 | 0.010 | <0.001 |  | 0.103 | 0.010 | <0.001 |  | 0.099 | 0.010 | <0.001 |
| SC5 | 1.054 | 0.090 | <0.001 |  | 1.026 | 0.093 | <0.001 |  | 0.968 | 0.093 | <0.001 |  | 0.130 | 0.009 | <0.001 |  | 0.135 | 0.009 | <0.001 |  | 0.129 | 0.009 | <0.001 |
| **Usual activities** |  |  |  |  |  |  |  |  |  |  |  |  |  |  |  |  |  |  |  |  |  |  |  |
| UA2 | 0.254 | 0.077 | 0.001 |  | 0.280 | 0.079 | <0.001 |  | 0.326 | 0.088 | <0.001 |  | 0.042 | 0.009 | <0.001 |  | 0.042 | 0.010 | <0.001 |  | 0.036 | 0.009 | <0.001 |
| UA3 | 0.111 | 0.086 | 0.195 |  | 0.084 | 0.087 | 0.338 |  | 0.120 | 0.088 | 0.173 |  | 0.034 | 0.009 | <0.001 |  | 0.032 | 0.010 | 0.001 |  | 0.030 | 0.010 | 0.002 |
| UA4 | 0.818 | 0.089 | <0.001 |  | 0.837 | 0.091 | <0.001 |  | 0.846 | 0.089 | <0.001 |  | 0.122 | 0.010 | <0.001 |  | 0.124 | 0.010 | <0.001 |  | 0.121 | 0.010 | <0.001 |
| UA5 | 1.167 | 0.099 | <0.001 |  | 1.195 | 0.102 | <0.001 |  | 1.208 | 0.100 | <0.001 |  | 0.178 | 0.009 | <0.001 |  | 0.184 | 0.010 | <0.001 |  | 0.174 | 0.010 | <0.001 |
| **Pain/discomfort** |  |  |  |  |  |  |  |  |  |  |  |  |  |  |  |  |  |  |  |  |  |  |  |
| PD2 | 0.439 | 0.082 | <0.001 |  | 0.405 | 0.085 | <0.001 |  | 0.386 | 0.091 | <0.001 |  | 0.029 | 0.009 | <0.001 |  | 0.029 | 0.010 | 0.002 |  | 0.021 | 0.009 | 0.018 |
| PD3 | 0.562 | 0.088 | <0.001 |  | 0.538 | 0.090 | <0.001 |  | 0.500 | 0.090 | <0.001 |  | 0.053 | 0.009 | <0.001 |  | 0.053 | 0.010 | <0.001 |  | 0.050 | 0.010 | <0.001 |
| PD4 | 2.164 | 0.101 | <0.001 |  | 2.141 | 0.103 | <0.001 |  | 2.142 | 0.102 | <0.001 |  | 0.287 | 0.010 | <0.001 |  | 0.295 | 0.010 | <0.001 |  | 0.300 | 0.011 | <0.001 |
| PD5 | 3.351 | 0.132 | <0.001 |  | 3.313 | 0.135 | <0.001 |  | 3.323 | 0.132 | <0.001 |  | 0.435 | 0.010 | <0.001 |  | 0.452 | 0.011 | <0.001 |  | 0.446 | 0.012 | <0.001 |
| **Anxiety/depression** |  |  |  |  |  |  |  |  |  |  |  |  |  |  |  |  |  |  |  |  |  |  |  |
| AD2 | 0.509 | 0.084 | <0.001 |  | 0.553 | 0.086 | <0.001 |  | 0.540 | 0.093 | <0.001 |  | 0.069 | 0.009 | <0.001 |  | 0.069 | 0.010 | <0.001 |  | 0.063 | 0.009 | <0.001 |
| AD3 | 1.110 | 0.092 | <0.001 |  | 1.149 | 0.094 | <0.001 |  | 1.140 | 0.094 | <0.001 |  | 0.156 | 0.010 | <0.001 |  | 0.158 | 0.010 | <0.001 |  | 0.161 | 0.010 | <0.001 |
| AD4 | 2.177 | 0.112 | <0.001 |  | 2.200 | 0.116 | <0.001 |  | 2.165 | 0.116 | <0.001 |  | 0.297 | 0.009 | <0.001 |  | 0.304 | 0.010 | <0.001 |  | 0.301 | 0.010 | <0.001 |
| AD5 | 3.057 | 0.125 | <0.001 |  | 3.111 | 0.130 | <0.001 |  | 3.059 | 0.127 | <0.001 |  | 0.408 | 0.009 | <0.001 |  | 0.422 | 0.010 | <0.001 |  | 0.416 | 0.010 | <0.001 |

Note: For c-TTO modelling, we tested the c-TTO data excluding 2 participants (2 non-traders). We obtained the same results as those shown in the manuscript (therefore the results were not shown here).

For DCE modelling (model 1-3), we tested 3 DCE data sets, including 1. removing participants with flat lining behavior (such as AAAAAAA or ABABABA), n=22; 2. removing respondents with shorter response time (<=200 seconds), n=65; 3. removing 10 DCE mild health state vs mild health state pairs.

For hybrid modelling (model 4-6), we used the c-TTO data (all) and DCE data without 10 mild pairs as an example to demonstrate here.

Figure S1. c-TTO value by level of sum score, after excluded values flagged by feedback model (n=6874)

Figure S2. Relative preference for health state A versus B by difference in level of sum score for discrete choice experiment tasks

Figure S3. Scatterplots of the predicted values of the GLS model versus observed values of c-TTO
